# Adverse Pregnancy Outcomes in Women with Gestational Diabetes Using Different Diagnostic Criteria: A Study from the Northern Adriatic Region of Croatia

**DOI:** 10.3390/medicina61122218

**Published:** 2025-12-16

**Authors:** Iva Plisic, Oleg Petrovic, Gabrijela Sopta Primorac, Ksenija Bazdaric, Marko Klaric, Dubravka Jurisic-Erzen

**Affiliations:** 1 Faculty of Medicine, University of Rijeka, Brace Branchetta 20, 51000 Rijeka, Croatia; 2 Department of Obstetrics and Gynecology, Clinical Hospital Centre Rijeka, Kresimirova 42, 51000 Rijeka, Croatia; 3 Department of Basic Medical Sciences, Faculty of Health Studies, University of Rijeka, Viktora Cara Emina 5, 51000 Rijeka, Croatia; 4 Department of Endocrinology and Diabetology, Clinical Hospital Centre Rijeka, Kresimirova 42, 51000 Rijeka, Croatia

**Keywords:** diagnosis, gestational diabetes, hyperglycemia, pregnancy complications

## Abstract

*Background and Objectives*: Gestational diabetes mellitus (GDM) is a complex pregnancy condition that carries substantial risks for adverse pregnancy outcomes. Following the implementation of universal diagnostic criteria in our clinical practice, this study was undertaken to assess their applicability and to determine whether locally conducted clinical studies are beneficial before adopting globally applicable criteria. By retrospectively analyzing parameters relevant to GDM from medical records, we aimed to determine the suitability of existing diagnostic criteria for our population, taking into account distinct socioeconomic, demographic, and genetic factors, and to assess the validity of alternative criteria. *Materials and Methods*: We used data from 2183 pregnant women who underwent 75 g-OGTT between 24 and 28 weeks of pregnancy. Results of the plasma glucose (PG) measurements were used to assign women into four diagnostic groups: diagnosed and treated by IADPSG criteria, diabetes mellitus in pregnancy identified according to WHO-2006 criteria, identified according to CDA-2013 criteria, and identified according to Tomic et al. criteria, based on a study on our population. Pregnancy outcomes were extracted from medical records. *Results*: The prevalence of GDM was 18.7% by IADPSG criteria, comparable to published data. Adverse pregnancy outcomes were consistently more frequent in GDM groups across all diagnostic systems (46.6–80% versus 33.9–35.9% in non-GDM). Maternal BMI ≥ 25 kg/m^2^ was also associated with having large-for-gestational-age (LGA) neonates, contributing to the influence of hyperglycemia. Excessive gestational weight gain was a predictor of complications such as macrosomia and cesarean delivery. *Conclusions*: Before adopting universal GDM diagnostic criteria, population-specific studies are valuable to balance detection rates and clinical accuracy.

## 1. Introduction

Pregnancy is a physiological condition associated with relative insulin resistance, primarily mediated by placental hormones. This metabolic shift, alongside other changes, facilitates the adequate supply of glucose to the fetus [1]. During pregnancy, the pancreas in women responds to these changes by increasing insulin secretion. However, when the insulin levels fail to meet the requirements, gestational diabetes mellitus (GDM) may develop. The estimated global prevalence of gestational diabetes, according to the criteria outlined by the International Association of Diabetes and Pregnancy Study Groups (IADPSG), is approximately 17% [2]. Over recent decades, there has been a notable increase in the prevalence of GDM, attributed to factors such as delayed motherhood and pregestational obesity [3].

Gestational diabetes mellitus is a condition that affects both maternal and fetal health and includes short-term as well as long-term consequences [4]. Pregnancy complications most commonly associated with GDM include having a large-for-gestational-age (LGA) infant and fetal macrosomia, which can lead to birth trauma and necessitate operative delivery. Having a small-for-gestational-age (SGA) neonate can also be a GDM-related complication resulting from vascular and endothelial dysfunction, placental insufficiency, or excessively restrictive glycemic control, all of which reduce the availability of essential nutrients and oxygen required for normal fetal growth and development [5]. Additionally, hypertensive disorders in pregnancy may occur, potentially resulting in preterm birth and neonatal respiratory and metabolic complications. Women who develop GDM face an elevated risk of later developing type 2 diabetes and cardiovascular conditions. Furthermore, neonates born to mothers with GDM are at a higher risk of developing adolescent obesity, glucose intolerance, hypertension, and metabolic syndrome [6]. Literature shows that while the diabetic condition itself carries the risk for developing complications, it is clinically relevant to evaluate glycemic control [7].

Several studies have investigated the impact of different GDM diagnostic criteria on adverse pregnancy outcome prevalence, but results remain unclear [4,8]. Some of them suggest an increased prevalence of GDM when IADPSG criteria are used, yet without significant differences in the prevalence of adverse pregnancy outcomes [9]. The question that arises is whether there are appropriate strategies and criteria for GDM diagnosis, since the harm and benefit of reducing the threshold of diagnostic criteria impacts pregnancy outcomes, women’s psychological well-being, and health costs. There is emerging evidence that GDM criteria may not be universally applicable [10,11].

It remains unclear to what extent lowering OGTT plasma glucose diagnostic thresholds reflects the probability of adverse pregnancy outcomes and whether ethnic background, obesity, and excessive gestational weight gain may influence these relationships. The aim of our study is to estimate the prevalence of the most common adverse outcomes associated with GDM among Croatian women identified by four different criteria: IADPSG [2], WHO-2006 [12], CDA [13], and Tomic et al. [14]. While the first three diagnostic systems are well established, the fourth, by Tomic et al., was based on our local population in a study that evaluated maternal glycemia thresholds linked to specific adverse outcomes in 1002 high-risk pregnancies. Participants underwent a modified 75 g oral glucose tolerance test (OGTT), and results were analyzed alongside pregnancy outcomes.

## 2. Materials and Methods

### 2.1. Study Design and Population

This retrospective cohort study utilized data from 2183 pregnant women who underwent an oral glucose tolerance test (OGTT) between 24 and 28 weeks of pregnancy and delivered at the Perinatology Department of the Clinical Medical Center Rijeka from 2014 to 2018.

The OGTT was conducted in accordance with standard laboratory protocols, with glucose levels measured in the fasting state and at 60 and 120 min after a 75 g glucose load. During data collection, the diagnosis of GDM was based on the IADPSG criteria. All patients were provided with medical advice regarding lifestyle modifications and dietary changes.

Participants with pre-existing type 1 or type 2 diabetes, those with incomplete demographic, pregnancy complication, or delivery information, and those with multifetal pregnancies were excluded from the study.

Data on maternal characteristics, including age, number of prior deliveries, pre-pregnancy weight and height, gestational weight gain, smoking status, and family history of type 2 diabetes mellitus, were extracted from hospital admission records. Pre-pregnancy body mass index (BMI) was calculated and categorized according to the WHO International Classification as follows: underweight (<18.5 kg/m^2^), normal weight (18.5–24.9 kg/m^2^), overweight (25–29.9 kg/m^2^), and obese (≥30 kg/m^2^) [15].

### 2.2. Obstetric and Neonatal Outcomes

We manually gathered data related to pregnancy complications potentially associated with GDM. These complications included hypertension in pregnancy greater than or equal to 140/90 mmHg, with or without proteinuria [16]. We also recorded cases of polyhydramnios, diagnosed through ultrasound examination or clinical observation during labor [17]. Pregnancy outcomes included macrosomia, defined as birthweight exceeding 4000 g [18]. Additionally, we considered infants with birthweight above the 90th percentile as large-for-gestational-age (LGA), as well as those with birthweight below the 5th and 10th percentiles as small-for-gestational-age (SGA) for our specific population [19]. Operative deliveries due to cephalopelvic disproportion [20], hyperbilirubinemia (excluding cases of Rhesus isoimmunization), neonatal hypoglycemia (defined as blood glucose levels below 2.2 mmol/L, excluding intrauterine growth restriction), and the need for neonatal intensive care unit (NICU) admission lasting longer than 24 h were also included as outcomes.

### 2.3. Main Exposure and Covariates

The main exposure variable was GDM; we applied different diagnostic cut-offs to the same diagnostic OGTT. Based on the results, women were retrospectively assigned to the following and partly overlapping diagnostic groups:

1. GDM diagnosed and treated according to the IADPSG criteria (fasting plasma glucose ≥ 5.1 mmol/L and/or 1 h plasma glucose ≥ 10.0 mmol/L and/or 2 h plasma glucose ≥ 8.5 mmol/L).
2. Diabetes mellitus in pregnancy retrospectively identified according to WHO-2006 criteria (fasting plasma glucose ≥ 7.0 mmol/L and/or 2 h plasma glucose ≥ 11.1 mmol/L).
3. GDM retrospectively identified according to CDA-2013 criteria (fasting plasma glucose ≥ 5.3 mmol/L and/or 1 h plasma glucose ≥ 10.6 mmol/L and/or 2 h plasma glucose ≥ 9.0 mmol/L).
4. GDM retrospectively identified according to Tomic et al. [14] criteria, based on a study of our population (1 h plasma glucose ≥ 7.9 mmol/L and 2 h plasma glucose ≥ 7.5 mmol/L).

Comparisons were made between groups that were not mutually exclusive, meaning some participants were included in more than one group.

### 2.4. Statistical Analyses

Categorical data are presented as frequencies (*n*) and relative frequencies (%) and tested with the χ^2^ square test and the test of proportions. Quantitative data are presented as median (C) and interquartile ranges (25–75 percentile). Normality of data distribution was tested with the Kolmogorov–Smirnov test.

The multivariate analysis of predictors of macrosomia and cesarean section was carried out using logistic regression analysis. The Enter method was used for all logistic regression analyses, synchronously evaluating all included independent variables. Candidate variables for the multivariable logistic regression model were selected based on clinical relevance and prior evidence in the literature [21]. Variables with *p* < 0.20 in univariate analysis were included in the analysis and assessed for multicollinearity by calculating the variance inflation factor (VIF). All predictors were significant in univariate analysis, and VIF was < 1.6 for all predictors in both logistic regression analyses [22].

The level of statistical significance was set to *p* < 0.05, and all odds ratios and confidence intervals were given at this level.

MedCalc Statistical Software version 16.2.1 (MedCalc Software bvba, Ostend, Belgium; https://www.medcalc.org; 2016) and Jamovi (version 2.6, Sydney, Australia; https://www.jamovi.org) programs were used for statistical analyses.

### 2.5. Ethics

The study was conducted in accordance with the Declaration of Helsinki and approved by the Faculty of Medicine, University of Rijeka Ethics Committee (protocol code 007-08-22-01/77, URBr 2170-24-04-3/1-22-3, 29 November 2022), as well as the Ethics Committee of Clinical Hospital Center Rijeka (protocol code 003-05/20-1/24; URBr 2170-29-02/1-20-2, 25 February 2020) and the Ethics Committee of the Health center of Primorje—Gorski Kotar County (protocol code 01-7/3-2-20, 10 November 2020).

Informed consent was waived with the approval of the Ethics Committee, as the study was conducted retrospectively, using data exclusively obtained from hospital records. All procedures complied with bioethical standards, ensuring the privacy of participants and the confidentiality of their data. The study adhered to the four fundamental bioethical principles and their derivatives, in line with relevant regulatory documents. No interventions were performed on participants, as conducting studies involving pregnant women would raise ethical concerns.

## 3. Results

### 3.1. Study Sample Characteristics

Characteristics of the sample are presented in Table 1. There were a total of 2183 Caucasian pregnant women in this study, with a median age of 31 (25th–75th percentile: 28–34) years. The median BMI of patients was in the normal weight range, 22.7 (25th–75th percentile: 20.8–25.6) kg/m^2^, but there were ~30% of overweight and obese women. Around 15% of women were current smokers. The median gestational age at delivery was 40 (25th–75th percentile: 39–40) weeks. Eleven percent (*n* = 245) of the neonates were large-for-gestational-age. Thirty-six percent of women had specific complications, and 286 (13.1%) had cesarean sections.

### 3.2. Pregnancy Outcomes Based on Four Diagnostic Criteria

Maternal characteristics based on four diagnostic criteria for glucose tolerance are presented in Table 2. There were a total of 408 (18.7%) women diagnosed by the IADPSG criteria, 15 (0.7%) by the WHO diabetes mellitus in pregnancy criteria, 237 (10.9%) by the CDA criteria, and 183 (8.4%) by the Tomic et al. criteria. Statistical significance (*p*) for the WHO group was not calculated due to the small sample size.

Patients in all groups diagnosed with GDM were significantly older, by 1 to 5 years in all groups (all *p* < 0.05). Pre-pregnancy BMI was also higher (2.0–8.9 points) in all groups diagnosed as GDM (all *p* < 0.05). There were no differences in the frequency of smoking among groups (all *p* > 0.005). There were significantly more primiparous women in non-diabetic groups according to all criteria, except for Tomic et al.

Pregnancy outcomes based on four diagnostic criteria for glucose tolerance are presented in Table 3. All groups of women with GDM, except for the Tomic et al. criteria, had a significantly higher rate of cesarean section. As expected, macrosomia was more frequent in all GDM groups (all *p* < 0.05). All groups of women with GDM had a higher relative frequency of large-for-gestational-age (LGA) neonates compared to the non-GDM group (all *p* < 0.05) and a higher rate of complications (all *p* < 0.05).

**Figure 1:**
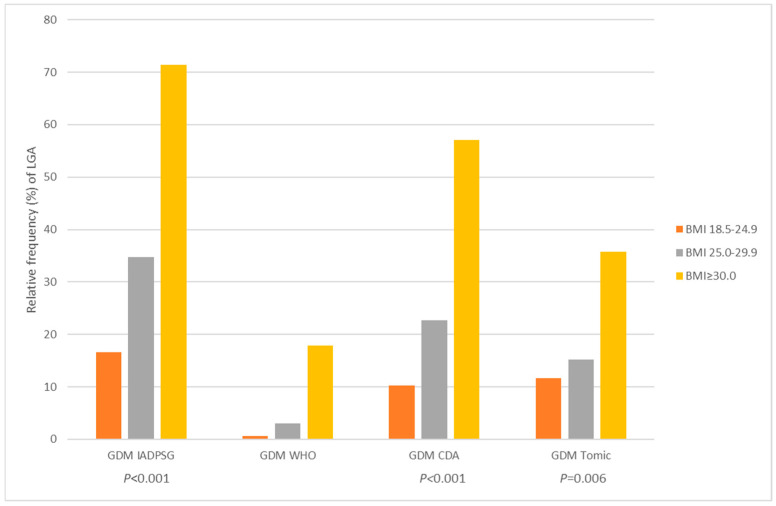
Relative frequency of large-for-gestational-age (LGA) by gestational diabetes mellitus (GDM) status according to four different criteria, in normal weight, overweight, and obese women (*n* = 239).

We analyzed 245 women with large-for-gestational-age neonates, for whom we related BMI categories according to different GDM criteria and excluded women with BMI < 18.5 kg/m^2^ (*n* = 6). Relative frequency (%) of LGA differed in relation to BMI according to different GDM criteria (all *p* < 0.05) and was most frequent in the BMI ≥ 30 kg/m^2^ group (Figure 1).

**Figure 2:**
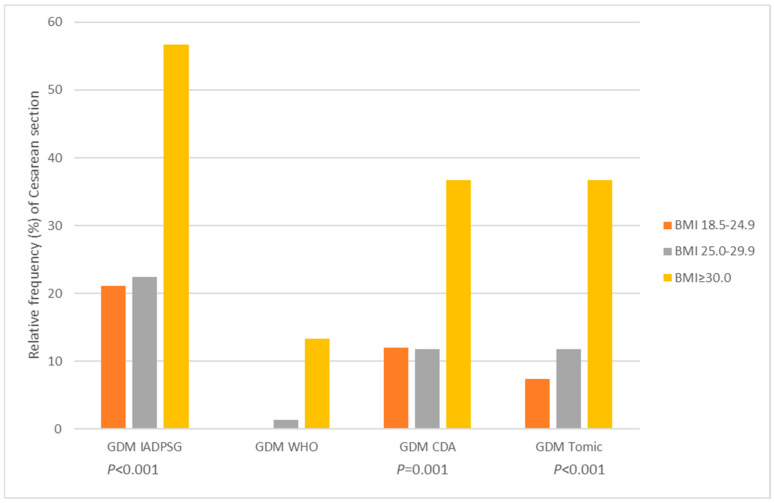
Relative frequency of cesarean section by gestational diabetes mellitus (GDM) status according to four different criteria, in normal weight, overweight, and obese women (*n* = 281).

We have analyzed 286 women with cesarean section, in which we related BMI categories according to different GDM criteria and excluded women with BMI < 18.5 kg/m^2^ (*n* = 5), resulting in a total of 281 women. The relative frequency of cesarean sections was different in relation to BMI according to different GDM criteria (all *p* < 0.05) and was most frequent in the BMI ≥ 30 kg/m^2^ group (Figure 2).

### 3.3. Prediction of Pregnancy Outcomes

Two logistic regressions were performed. The first logistic regression was performed to build a model of macrosomia prediction (birthweight ≥ 4000 g) with parity and gestational age as covariates (Table 4). The logistic regression model of macrosomia was significant (*p* < 0.001), had acceptable discrimination (AUC = 0.72), and had a Nagelkerke R^2^ of 0.13. Gestational diabetes diagnosed using the Tomic et al. criteria was associated with twice the odds of macrosomia (OR = 2.02, 95% CI: 1.30–3.15, *p* = 0.002). Excessive gestational weight gain was also positively associated (OR = 2.00, 95% CI: 1.48–2.69, *p* < 0.001) with macrosomia. Smoking was negatively associated with macrosomia (OR = 0.49, 95% CI: 0.33–0.73, *p* < 0.001). Other predictors, including BMI, mother’s age, and GDM classified using IADPSG or CDA criteria, were not statistically significantly associated with macrosomia.

The second logistic regression model examined predictors of cesarean section due to cephalopelvic disproportion (Table 5). The logistic regression model was significant (*p* < 0.001), had good discrimination (AUC = 0.78), and had a Nagelkerke R^2^ of 0.08. Excessive gestational weight gain was a significant predictor, and the odds ratio for a cesarean section due to cephalopelvic disproportion was 1.83 (95% CI: 1.14–2.93). Adiposity was also a significant predictor, and the odds ratio for pre-pregnancy BMI ≥ 30 kg/m^2^ was 1.94 (95% CI: 1.08–3.51). Parity and gestational age were significant covariates.

## 4. Discussion

Gestational diabetes mellitus, as a significant complication of pregnancy, is a complex diagnostic issue with no simple solution. Although many professional organizations have developed their own screening and diagnostic approaches, none of them seem to be universally applicable. Genetic, epidemiological, and risk factor variations across diverse global populations make standardized diagnostic criteria a challenge, and there is still no consensus on the most appropriate criteria to use. In this study, we aimed to evaluate the diagnostic strength of various diagnostic systems within our population, with a particular focus on outcome-based assessment through specific adverse pregnancy outcomes. While studies like this have been conducted in different local settings [23,24,25], none, to the best of our knowledge, have been conducted within our local population, which has a relatively homogenous ethnic structure and may differ from previously studied international multicenter cohorts in lifestyle, age distribution, and socioeconomic status. Local Mediterranean dietary habits and environmental influences could affect metabolic characteristics, highlighting the importance of evaluating the GDM diagnostic criteria in this specific context.

The prevalence of GDM in our population using the IADPSG criteria was 18.7%, similar to that reported in the literature [2], indicating that our study population was appropriately selected and representative of the population. The *p*-value for the WHO group was not calculated due to the small sample size; the results are presented but interpreted with caution. All pregnancy complications, except SGA, occurred more frequently in the GDM groups, with most showing statistically significant differences across the diagnostic criteria.

Hypertension in pregnancy was significantly more common in GDM groups than in non-GDM groups for all criteria. Polyhydramnios, macrosomia, and LGA as outcomes were also more frequent among women with GDM for all criteria. In contrast, SGA was less common in GDM groups, despite being a recognized potential complication. This suggests that while GDM carries a burden of adverse outcomes, the specific issue of SGA births may not be more frequent in GDM pregnancies, as already reported in the literature [26].

The total cesarean section rate was significantly higher in GDM groups for all criteria except Tomic et al. Interestingly, cesarean deliveries due to cephalopelvic disproportion did not reach statistical significance, likely because multiple indications for operative delivery were often recorded, making cephalopelvic disproportion underreported in medical records.

Neonatal hypoglycemia (<2.2 mmol/L) was rare overall and reached statistical significance only in the GDM group defined by IADPSG criteria. Hyperbilirubinemia was significantly more frequent in all GDM groups. Although neonatal intensive care unit (NICU) admission was more common in GDM groups, the difference was not statistically significant.

Overall, the total presence of adverse pregnancy outcomes was significantly higher in GDM groups for all diagnostic criteria, with notable percentage differences compared with non-GDM groups. In the non-GDM groups, the rate varied between 33.9% and 35.9%, whereas in the GDM groups it was 46.6%, 80%, 49.8%, and 51.9% for the IADPSG, WHO, CDA, and Tomic et al. criteria, respectively. The results indicate that criteria with higher glucose thresholds are associated with lower detection rates and, as expected, higher rates of complications. The WHO criteria for diabetes in pregnancy, which have the highest diagnostic thresholds, were associated with the highest complication rate but identified only a few of the GDM patients. Presenting these results is primarily to illustrate the “historical” approach to gestational diabetes, which seems to have been associated with substantial underdiagnosis, while the relatively few cases that were identified experienced more severe complications—one of the reasons these criteria were later abandoned in clinical practice. The Tomic et al. criteria, by contrast to the first three, consider elevated second and third glucose values but not the first, and in our analyses, they were associated with a lower GDM detection rate of 8.4% yet higher complication rates than both the IADPSG and CDA criteria. However, the clinical implications of adopting such criteria are twofold. On one hand, they may help direct medical care towards more complex cases and alleviate the psychological burden for individuals at relatively low risk of hyperglycemia-related adverse outcomes. On the other hand, it would leave the condition underdiagnosed and some patients untreated. Finding an appropriate balance between adequate prevention and avoiding unnecessary intervention remains an important and complex issue. Management strategies should be adapted to the specific characteristics and needs of each population. In certain contexts, broader inclusion of treatment cases may be warranted, taking into consideration available resources and the objective of maximizing safety. Conversely, in settings with more limited resources, a focused approach targeting smaller, well-defined groups may be preferable to optimize clinical outcomes.

Although specific adverse pregnancy outcomes are associated with GDM, none of them are exclusive to the condition, which complicates the diagnostic process. Both maternal pregestational BMI and hyperglycemia contribute to excessive fetal growth, with BMI often having greater influence than glycemia, according to the literature [27]. In our study, women with BMI ≥ 30 kg/m^2^ had higher rates of large-for-gestational-age (LGA) neonates across all GDM diagnostic groups (Figure 1, all *p* < 0.05), which confirms the link between maternal weight and neonatal birthweight. Although LGA is one of the most widely recognized GDM-related outcomes [28], authors have reported that BMI remains a stronger predictor except at the highest glucose levels. Most LGA cases occur in women with normal glycemia, indicating that maternal weight or weight gain may play a predominant role [29]. Parental body size and birthweight are also influenced by genetic and environmental factors, which are difficult to quantify and likely confound such associations. In our study (Table 4), the macrosomia prediction model showed that excessive gestational weight gain is an independent predictor, while maternal weight did not reach statistical significance. These findings are somewhat in line with those reported in the literature [30].

Primary cesarean section is similarly affected by both maternal BMI and glycemia [31]. In our data, the rates of cesarean section in women with GDM stratified by BMI differed statistically between groups (Figure 2, all *p* < 0.05) and occurred more frequently in women with BMI ≥ 30 kg/m^2^. When tested as an independent predictor for cesarean delivery due to cephalopelvic disproportion, both maternal weight and weight gain were significant predictors.

The main limitation of our study is that it was carried out retrospectively. We are also aware that the IADPSG criteria were used for both diagnosis and management and that an independent gold standard for gestational diabetes mellitus was not available. All women were diagnosed and managed using IADPSG criteria, with lifestyle and dietary interventions as needed. This may bias retrospective reclassification, as women considered “non-GDM” under other diagnostic systems could still have received treatment, potentially lowering their complication rates. But as the evidence in the literature suggests, there is a residual risk of complications even when GDM is treated; while treatment substantially reduces the risk, it does not eliminate it, and the risk remains higher than in women without GDM [32]. One of the main strengths of the present study is the sample size, which was adequate for making statistically valid conclusions for our local Caucasian population and well-defined clinical outcomes as endpoints. Unlike many studies, participants were not selected based on high risk, as an OGTT was offered to all patients during pregnancy. All the participants gave birth in the same clinical hospital center, and therefore, all the specific complications were recorded in the same way. We had sufficient data to compare various GDM diagnostic systems and used adequate statistical methods.

## 5. Conclusions

As well as many authors, we believe that before introducing diagnostic criteria for GDM into clinical practice within a given population, a preliminary study should be conducted to determine the optimal (regional or national) criteria, taking into account epidemiological, ethnic, and sociocultural characteristics of the population. Therefore, consideration should be given to the detection rate of true GDM or hyperglycemia cases, the psychological impact on the pregnant woman, and the potential burden on the healthcare system. Although lifestyle and dietary changes due to GDM diagnosis could have a lifelong beneficial effect on a patient, cost-effectiveness should also be taken into account. Our findings may provide insight into how population-specific factors can influence the performance of established criteria; our intention was not to give preference to any of the diagnostic systems examined but rather to objectively present their diagnostic values from a clinical perspective. Recent literature and the results of our study show that the task of detecting and establishing highly effective, ideal universal diagnostic criteria has still not been achieved (or completed). Therefore, with our research, we aim to encourage numerous experts and scientists around the world to fully devote themselves to this professional goal in their future well-planned, organized, and well-conducted studies.

## Figures and Tables

**Table 1:** Sociodemographic data of the whole sample.

Variable	C (25–75) or *n* (%)
Maternal age (years)	31 (28–34)
Pre-pregnancy BMI (kg/m^2^)	22.7 (20.8–25.6)
Underweight < 18.5 kg/m^2^, *n* (%)	96 (4.4)
Normal weight 18.5–24.9 kg/m^2^, *n* (%)	1447 (66.3)
Overweight 25–29.9 kg/m^2^, *n* (%)	449 (20.6)
Obese ≥ 30.0 kg/m^2^, *n* (%)	191 (8.7)
Current smoker, *n* (%)	326 (14.9)
Fasting plasma glucose (mmol/L)	4.7 (4.4–5.0)
1 h plasma glucose (mmol/L)	7.1 (5.9–8.5)
2 h plasma glucose (mmol/L)	5.7 (4.8–6.7)
Gestational age at delivery (weeks)	40.0 (39.0–40.0)
Birthweight (g)	
Male neonates	3590 (3260–3920)
Female neonates	3440 (3150–3720)
LGA, 90th percentile, *n* (%)	245 (11.2)
SGA, 5th percentile, *n* (%)	104 (4.8)
SGA, 10th percentile, *n* (%)	211 (9.7)
Preterm birth, *n* (%)	93 (4.3)
Hypertension in pregnancy, *n* (%)	92 (4.2)
Caesarean section, *n* (%)	286 (13.1)
Complications, overall, *n* (%)	791 (36.2)

Legend: C = median value, 25–75 = 25th–75th percentile values, BMI = body mass index, LGA = large-for-gestational-age, and SGA = small-for-gestational-age.

**Table 2:** Maternal characteristics based on four diagnostic criteria for GDM.

Variable	IADPSG Criteria			WHO Criteria			CDA Criteria			Tomic et al. Criteria		
	Non-GDM *n* = 1775 (81.3%)	GDM *n* = 408 (18.7%)	*p*	Non-GDM *n* = 2168 (99.3%)	GDM *n* = 15 (0.7%)	*p*	Non-GDM *n* = 1946 (89.1%)	GDM *n* = 237 (10.9%)	*p*	Non-GDM *n* = 2000 (91.6%)	GDM *n* = 183 (8.4%)	*p*
	C (25–75) or *n* (%)			C (25–75) or *n* (%)			C (25–75) or *n* (%)			C (25–75) or *n* (%)		
Maternal age	31.0 (28.0–34.0)	32.0 (29.0–35.5)	<0.001	31.0 (28.0–34.0)	36.0 (32.3–38.5)	/	31.0 (28.0–34.0)	33.0 (29.0–36.0)	<0.001	31.0 (28.0–34.0)	33.0 (30.3–36.0)	<0.001
Pre-pregnancy BMI (kg/m^2^)	22.3 (20.5–25.0)	24.8 (22.1–28.8)	<0.001	22.6 (20.7–25.5)	31.5 (27.1–32.9)	/	22.3 (20.5–25.0)	25.1 (22.6–29.1)	<0.001	22.5 (20.7–25.3)	24.5 (21.9–28.2)	<0.001
Underweight	84 (4.7)	12 (2.9)	<0.001	96 (4.4)	0 (0.0)	/	94 (4.8)	2 (0.8)	<0.001	93 (4.6)	3 (1.6)	<0.001
Normal weight	1243 (70.0)	204 (50.0)		1444 (66.7)	3 (20.0)		1332 (68.5)	115 (48.5)		1350 (67.5)	97 (53.0)	
Overweight	339 (19.1)	110 (27.0)		445 (20.5)	4 (26.7)		381 (19.6)	68 (28.7)		405 (20.3)	44 (24.0)	
Obesity	109 (6.1)	82 (20.1)		183 (8.4)	8 (53.3)		139 (7.1)	52 (21.9)		152 (7.6)	39 (21.3)	
Smoking, *n* = 326	268 (15.1)	58 (14.2)	0.608	324 (14.9)	2 (13.3)	/	289 (14.9)	37 (15.6)	0.685	303 (15.2)	23 (12.6)	0.466
Fasting PG	4.6 (4.4–4.8)	5.2 (5.1–5.4)	<0.001	4.6 (4.4–5.0)	5.9 (5.5–7.7)	/	4.6 (4.4–4.8)	5.4 (5.1–5.6)	<0.001	4.6 (4.4–4.9)	5.0 (4.6–5.4)	<0.001
1 h PG	6.6 (5.5–7.8)	9.1 (7.3–10.6)	<0.001	7.0 (5.9–8.4)	11.9 (11.4–12.9)	/	6.8 (5.7–8.1)	10.2 (8.3–11.2)	<0.001	6.8 (5.7–8.0)	10.1 (8.9–11.2)	<0.001
2 h PG	5.4 (4.6–6.3)	6.7 (5.6–8.4)	<0.001	5.7 (4.8–6.7)	11.5 (11.1–12.4)	/	5.5 (4.7–6.4)	7.3 (5.0–9.1)	<0.001	5.5 (4.7–6.3)	8.4 (7.9–9.5)	<0.001
Primipara, *n* = 1215	1008 (56.8)	207 (50.7)	0.028	1209 (55.8)	6 (40.0)	/	1103 (56.7)	112 (47.3)	0.003	1118 (55.9)	97 (53.0)	0.434

Legend: C (25–75) or *n* (%) = data are presented as median (25th–75th percentile) values or *n* (%), / = χ^2^ was not calculated due to small sample size, BMI = body mass index, GDM = gestational diabetes mellitus, PG = plasma glucose.

**Table 3:** Pregnancy outcomes based on four diagnostic criteria for GDM.

Variable	IADPSG Criteria			WHO Criteria			CDA Criteria			Tomic et al. Criteria		
	Non-GDM *n* = 1775 (81.3%)	GDM *n* = 408 (18.7%)	*p*	Non-GDM *n* = 2168 (99.3%)	GDM *n* = 15 (0.7%)	*p*	Non-GDM *n* = 1946 (89.1%)	GDM *n* = 237 (10.9%)	*p*	Non-GDM *n* = 2000 (91.6%)	GDM *n* = 183 (8.4%)	*p*
	C (25–75) or *n* (%)			C (25–75) or *n* (%)			C (25–75) or *n* (%)			C (25–75) or *n* (%)		
Complications, *n* = 791	601 (33.9)	190 (46.6)	<0.001	779 (35.9)	12 (80.0)	/	673 (34.6)	118 (49.8)	<0.001	696 (34.8)	95 (51.9)	<0.001
Maternal/Obstetric Outcomes												
Hypertension *n* = 92	52 (2.9)	40 (9.8)	<0.001	87 (4.0)	5 (33.3)	/	60 (3.1)	32 (13.5)	<0.001	71 (3.5)	21 (11.5)	<0.001
CS CPD, *n* = 136	107 (6.0)	29 (7.1)	0.416	133 (6.1)	3 (20.0)	/	117 (6.0)	19 (8.0)	0.228	120 (6.0)	16 (8.7)	0.142
CS, total, *n* = 286	214 (12.1)	72 (17.6)	0.001	281 (13.0)	5 (33.3)	/	244 (12.5)	42 (17.7)	0.034	253 (12.7)	33 (18.0)	0.058
Polyhydramnios *n* = 22	10 (0.6)	12 (2.9)	0.001	20 (0.9)	2 (13.3)	/	15 (0.8)	7 (3.0)	0.001	17 (0.9)	5 (2.7)	0.015
Fetal/Neonatal Outcomes												
Macrosomia, *n* = 343	260 (14.6)	83 (20.3)	0.003	336 (15.5)	7 (46.7)	/	291 (15.0)	52 (21.9)	0.032	298 (14.9)	45 (25.3)	<0.001
LGA, *n* = 245	177 (10.0)	68 (16.7)	<0.001	237 (10.9)	8 (53.3)	/	199 (10.2)	46 (19.4)	<0.001	208 (10.4)	37 (20.0)	<0.001
SGA, *n* = 211	174 (9.8)	37 (9.1)	0.651	210 (9.7)	1 (6.7)	/	190 (9.8)	21 (8.9)	0.657	196 (9.8)	15 (8.2)	0.482
Hypoglycemia, *n* = 8	3 (0.2)	5 (1.2)	<0.001	8 (0.4)	0 (0.0)	/	6 (0.3)	2 (0.8)	0.198	6 (0.3)	2 (1.1)	0.089
Hyperbilirubinemia, *n* = 234	170 (9.6)	64 (15.7)	<0.001	230 (10.6)	4 (26.7)	/	190 (9.8)	44 (18.6)	<0.001	202 (10.1)	32 (17.5)	0.002
NICU, *n* = 34	24 (1.4)	10 (2.5)	0.106	33 (1.5)	1 (6.7)	/	28 (1.4)	6 (2.5)	0.200	29 (1.5)	5 (2.7)	0.180

Legend: C (25–75) or *n* (%) = data are presented as median (25th–75th percentile) values or *n* (%), / = χ^2^ was not calculated due to small sample size, CS CPD = cesarean section due to cephalopelvic disproportion, CS = cesarean section, GDM = gestational diabetes mellitus, LGA = large-for-gestational-age, SGA = small-for-gestational-age, NICU = Neonatal Intensive Care Unit.

**Table 4:** Logistic regression models investigating contributions of macrosomia (*n* = 343).

Predictor	Odds Ratio	Lower 95% CI	Upper 95% CI	*p*
GDM based on IADPSG criteria	1.12	0.72	1.73	0.630
GDM based on CDA criteria	1.27	0.74	2.18	0.384
GDM based on Tomic et al. criteria	2.02	1.30	3.15	0.002
Pre-pregnancy BMI, overweight	1.13	0.84	1.52	0.433
Pre-pregnancy BMI, obese	1.25	0.83	1.87	0.290
Gestational weight gain, excessive	2.00	1.48	2.69	<0.001
Cigarette smoking	0.49	0.33	0.73	<0.001
Parity	0.78	0.68	0.90	<0.001
Gestational age at delivery	0.64	0.57	0.72	<0.001
Mother’s age	0.98	0.95	1.00	0.101

Legend: parity, gestational age at delivery, and mother’s age were covariates, 95% CI = 95% confidence interval, BMI = body mass index, GDM = gestational diabetes mellitus.

**Table 5:** Logistic regression models investigating independent contributions of cesarean section due to cephalopelvic disproportion (*n* = 136).

Predictor	Odds Ratio	Lower 95%CI	Upper 95%CI	*p*
GDM based on IADPSG criteria	1.17	0.58	2.36	0.655
GDM based on CDA criteria	0.75	0.33	1.73	0.505
GDM based on Tomic et al. criteria	0.70	0.36	1.36	0.292
Pre-pregnancy BMI, overweight	1.72	1.13	2.63	0.012
Pre-pregnancy BMI, obese	1.94	1.08	3.51	0.027
Gestational weight gain, excessive	1.83	1.14	2.93	0.012
Cigarette smoking	0.81	0.50	1.31	0.864
Parity	0.44	0.31	0.62	<0.001
Gestational age at delivery	1.29	1.10	1.50	<0.001
Mother’s age	1.03	0.99	1.01	0.118

Legend: parity, gestational age at delivery, and mother’s age were covariates, 95% CI = 95% confidence interval, BMI = body mass index, GDM = gestational diabetes mellitus.

## Data Availability

Data is available from the first author (IP) upon reasonable request.

## Abbreviations

The following abbreviations are used in this manuscript:

BMI	Body mass index
CDA	Canadian Diabetes Association
CS	Cesarean section
CS CPD	Cesarean section due to cephalopelvic disproportion
GDM	Gestational diabetes mellitus
IADPSG	International Association of Diabetes and Pregnancy Study Groups
LGA	Large-for-gestational-age
NICU	Neonatal intensive care unit
OGTT	Oral glucose tolerance test
PG	Plasma glucose
SGA	Small-for-gestational-age
WHO	World Health Organization
